# Editorial: New Trends in Connectomics

**DOI:** 10.1162/netn_e_00052

**Published:** 2018-06-01

**Authors:** Olaf Sporns, Danielle S. Bassett

**Affiliations:** Department of Psychological and Brain Sciences, Indiana University, Bloomington, IN, USA; Department of Bioengineering, University of Pennsylvania, Philadelphia, PA, USA; Department of Physics and Astronomy, University of Pennsylvania, Philadelphia, PA, USA; Department of Neurology, Hospital of the University of Pennsylvania, Philadelphia, PA, USA; Department of Electrical and Systems Engineering, University of Pennsylvania, Philadelphia, PA, USA

## Abstract

Connectomics is an integral part of network neuroscience. The field has undergone rapid expansion over recent years and increasingly involves a blend of experimental and computational approaches to brain connectivity. This Focus Feature on “New Trends in Connectomics” aims to track the progress of the field and its many applications across different neurobiological systems and species.

The idea that connections among neural elements are crucial for brain function has been central to modern neuroscience almost since its inception. Building on this idea, the emerging field of connectomics adds several new and important components. First, connectomics provides comprehensive maps of neural connections, with the ultimate goal of achieving complete coverage of any given nervous system. Second, connectomics delivers insights into the principles that underlie network architecture and uncovers how these principles support network function. These dual aims can be accomplished through the confluence of new experimental techniques for mapping connections and new network science methods for modeling and analyzing the resulting large connectivity datasets. Hence, connectomics naturally blends empirical and computational approaches to gain fundamentally new insights into structure and function of brain networks.

Connectomics continues to expand rapidly. Since the term “connectome” was first introduced in 2005 (Sporns, Tononi, & Kötter, 2005), the number of scientific articles devoted to connectomics has risen continuously (Figure 1). Since networks can be built on many spatial scales and with a variety of experimental techniques, there is a growing need to integrate across these different ways of constructing brain networks and to provide opportunities to exchange insights, data, and models. This need motivated the organization of a recent Keystone Symposium on connectomics with the explicit goal to foster scientific exchange among largely disconnected communities of researchers studying connectomes in different organisms at different scales with different measurement techniques. One central objective was to promote empirical and computational approaches that apply across scales, for example by leveraging the tools of network science (Bassett & Sporns, 2017). In March 2017, approximately 100 connectomics researchers gathered in Santa Fe (New Mexico) to exchange ideas and to discuss the future of the field. All presenters at the workshop were invited to submit their work to *Network Neuroscience*, to be gathered into a Focus Feature entitled “New Trends in Connectomics.”

**Figure 1.**
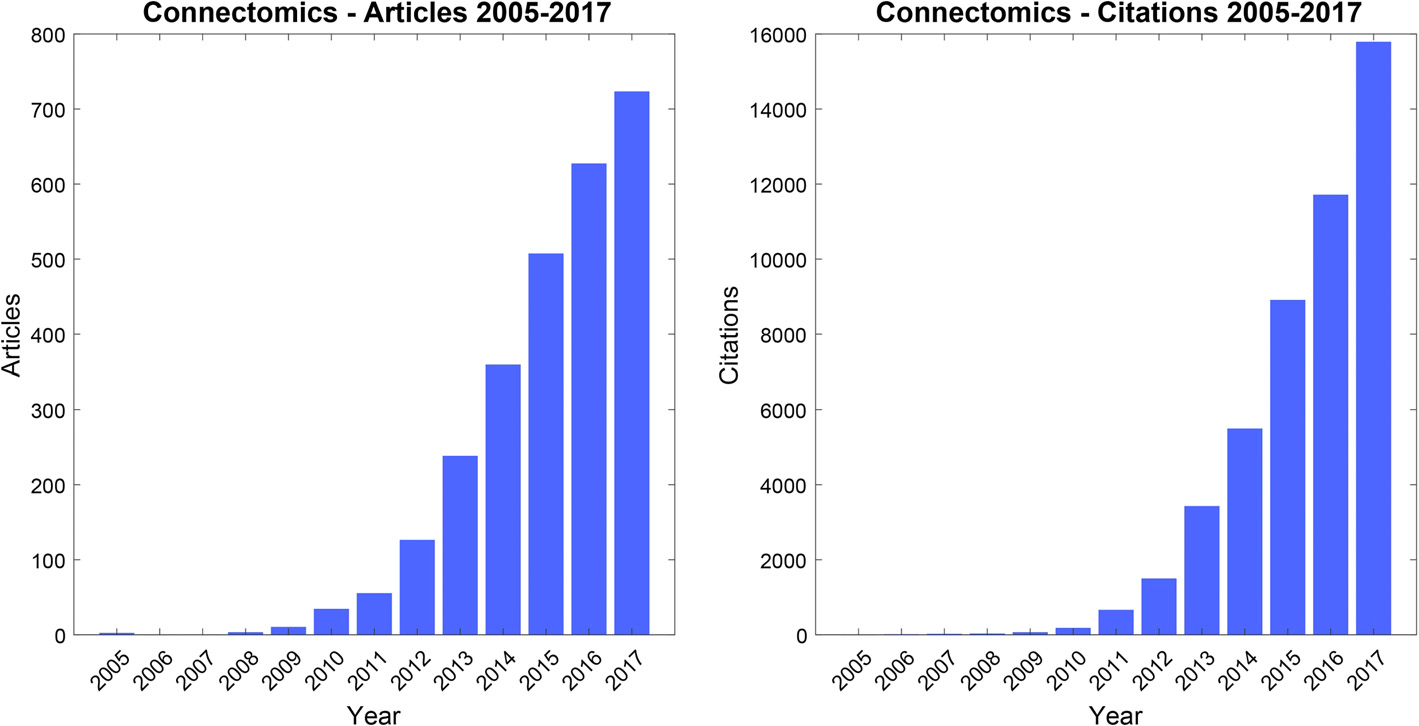
The growth of connectomics as indexed by the number of published articles and citations. Publication and citation counts were retrieved from Web of Science on March 12, 2018, using the search term “connectom*” in either topic or title. Through the end of 2017, a total of 2,684 articles were published, accruing a total of 47,725 citations. These counts likely underestimate the impact of connectomics, as many relevant articles do not reference the term “connectom*” in either topic or title.

The result, presented in this new issue of the journal, offers a panoramic overview of an emerging field, with papers that cover a range of techniques applied in different species, and that combine empirical data, modeling, and computation. Mattar, Thompson-Schill, and Bassett (2018) address how functional brain networks change over the course of learning, specifically those connections that link different network communities. Heitmann and Breakspear (2018) address the important role of nonlinear system dynamics in generating fluctuations in functional connectivity, an area of considerable interest in studies of spontaneous or “resting” brain activity. Miranda-Dominguez et al. (2018) use machine-learning techniques to demonstrate the heritability of “connectotyping”—the identification of individuals based on complex patterns of connectome-wide functional connectivity. Mills et al. (2018) apply functional connectomics to a human disorder (ADHD) and show that behavioral measures are associated with specific changes in the manner in which segregated functional systems interact with one another. Li et al. (2018) apply network science concepts to characterize the functional roles of epileptogenic zones in human electrophysiological data, and suggest that network-based methodologies may have clinical applications. Kesler, Acton, Rao, and Ray (2018) investigate structural and functional connectome network properties in a transgenic mouse model system designed to mimic the pathogenesis of Alzheimer’s disease. Kale, Zalesky, and Gollo (2018) investigate how the directionality of anatomical projections impacts the estimation of commonly used graph-theoretical attributes. Finally, Morgan, Achard, Termenon, Bullmore, and Vértes (2018) decompose functional brain networks into network motifs and use variations in motif frequency and composition to probe for generative processes underlying network formation.

Much discussion at the Keystone Symposium revolved around the issues that will occupy connectomics in the years to come. Clearly, continued improvements in measurement accuracy and coverage will give us improved connectivity maps, in a wider range of organisms and at various scales of resolution. With these developments will come an increasing need to design more advanced computational tools and theoretical frameworks to enable deeper insight into the fundamental principles of brain network architecture and function. And as maps and tools improve, the study of brain networks will continue to evolve, moving beyond descriptive accounts to models that incorporate a rich set of neurobiological mechanisms, address changes in network structure and dynamics, reveal the network basis of cognition and behavior, and enable targeted intervention, prediction, and control. *Network Neuroscience* will continue to serve as a prime forum for dissemination and discussion in this important field for many years to come.
